# Eosinophilic esophagitis associated with celiac disease in children

**DOI:** 10.1186/s13104-015-1256-z

**Published:** 2015-06-26

**Authors:** Rajmohan Dharmaraj, Karen Hagglund, Hernando Lyons

**Affiliations:** Department of Pediatrics, St. John Providence Children’s Hospital, Detroit, MI 48236 USA; Department of Biostatistics, St. John Providence Children’s Hospital, Detroit, MI 48236 USA; Department of Pediatric Gastroenterology, St. John Providence Childern’s Hospital, Wayne State University School of Medicine, Detroit, MI 48236 USA

## Abstract

**Background:**

Celiac disease (CD) and eosinophilic esophagitis (EoE) are distinct diseases of the gastrointestinal tract with specific clinico-pathological characteristics. Recent studies have found higher rates of EoE in patients with CD than in the general population. Our aim was to estimate the incidence of EoE among children who were diagnosed with CD over a 42-month period.

**Methods:**

The study included patients diagnosed with CD based on endoscopy and histopathological findings between January 2010 and June 2013. Histopathology reports of esophageal biopsies were reviewed to identify all cases of EoE. The patients’ presenting symptoms, laboratory evaluations, endoscopic and histopathological findings, treatments, and follow-ups were analysed.

**Results:**

Fifty-six patients with CD were identified, of whom six (10.7%) were diagnosed with both CD and EoE. Four of these patients presented with abdominal pain and diarrhea, two presented with failure to thrive, and three presented with food allergies. Endoscopic and histopathological changes typical of EoE were observed in all six patients. During follow-up, two patients showed significant improvement with the gluten-free diet and a proton-pump inhibitor (PPI). Two patients improved with the elimination diet and two patients were treated with topical corticosteroid therapy. Endoscopic appearance was normal in all children on follow-up endoscopy after treatment. Biopsy samples also showed resolution of the histologic features of EoE in all of the children.

**Conclusion:**

The incidence of EoE in our cohort of children with CD was 10.7%, which is higher than what has been reported for the general population. In all children undergoing upper gastrointestinal endoscopy for suspected CD, coexistence of EoE should be considered.

## Background

Eosinophilic esophagitis (EoE) and celiac disease (CD) are considered distinct immunologic diseases of the gastrointestinal tract with specific clinico-pathological characteristics. EoE is an emerging, chronic, inflammatory disorder of the esophagus. The incidence of EoE varies from 0.7 to 10 per 100,000 per person-year, and the prevalence ranges from 0.2 to 43 per 100,000 [1]. The incidence of EoE seems to be increasing in both adults and children, though it is still unclear whether this is a genuine phenomenon or attributable to an increasing awareness and detection of the disease. EoE presents with a range of diverse clinical symptoms. Symptoms in children vary by age with infants and young children typically presenting with feeding difficulties, reflux and failure to thrive. Vomiting, abdominal pain and regurgitation start to become apparent in school aged children and it is not until early adolescent and teenage years that children present with dysphagia and food impaction. Adults typically present with symptoms in the third or fourth decade of life, with the predominant symptoms being dysphagia, heart burn, food impaction and strictures. Diagnosis of EoE is based on the presence of symptoms in conjunction with finding of more than 15 eosinophils per high power field (HPF) in esophageal mucosal biopsy specimens, along with the exclusion of other diseases that cause esophageal eosinophilia [2]. The current diagnostic recommendations for EoE are that gastroesophageal reflux disease (GERD) be excluded by performing upper gastrointestinal endoscopy and biopsy while receiving proton-pump inhibitor (PPI) monotherapy. Although GERD was initially thought to cause only mild esophageal eosinophilia (<7 eosinophils/HPF), recent reports have shown that severe esophageal eosinophilia occurs and may resolve with acid-suppression monotherapy using PPI. The 2011 EoE consensus recommendations have utilized the term proton-pump inhibitor-responsive esophageal eosinophilia (PPI-REE) to describe this clinical and histological phenomenon [3]. However, the pathogenesis of this PPI-REE remains unclear. Studies from animal models and patients have suggested that EoE shares a clinical link with other atopic diseases and is caused by immune dysregulation secondary to allergic sensitization to dietary or aeroallergens. It is dominated by T-helper lymphocyte type 2 mediated eosinophil-predominant inflammation, with key contributions from mast cells, basophils, epithelial cells, and dendritic cells [4].

On the other hand, CD is an immune mediated disease of the small intestine, induced by the ingestion of gluten. This enteropathy may appear at any age and is characterized by a wide variety of clinical signs and symptoms. Among them, gastrointestinal presentations include chronic diarrhea, abdominal pain, weight loss, or failure to thrive in children; but extra-intestinal manifestations are also common [5]. CD affects roughly 1% of children, although many cases remain undiagnosed. Suspicion of CD should lead to antibody screening tests, and positive results should be followed by an intestinal biopsy for a definitive diagnosis [6]. Patients with CD are known to be at a higher risk for coexisting autoimmune diseases, including type 1 diabetes mellitus and autoimmune thyroiditis, but their risk of developing atopic diseases remains unclear [7, 8]. The only treatment currently available for CD is strict adherence to a gluten-free diet for life. Diagnosed, but untreated CD is associated with a significant increase in morbidity and mortality [9].

Recent case reports and cohort studies have suggested an association between CD and EoE in pediatric populations [10–15]. All the studies have used retrospectively assembled data or hospital admissions, however, which may have led to a bias in the results. Over a period of 42 months, we aimed to estimate the incidence of EoE in the cohort of children diagnosed as having CD at our center.

## Methods

### Participants

This is a prospective study that was conducted in the Pediatric Gastroenterology Clinic at St. John Providence Children’s Hospital, a tertiary referral center for the southeast Michigan community, upon approval by the St. John Hospital and Medical Center Institutional Review Board. Because this was an observational study, the institutional review board waived the requirement for informed consent. All pediatric patients with histologically confirmed CD who also had concurrent esophageal biopsies between January 2010 and June 2013 were included. Patients with inflammatory bowel disease were excluded from the study. Data on the following were collected during initial visit: age at diagnosis; gender; race; duration of symptoms; presenting symptoms; allergies; medications; and laboratory evaluations.

### Esophagogastroduodenoscopy and histology

All patients underwent upper gastrointestinal endoscopy using pediatric fiberoptic gastroscope at St. John Providence Children’s Hospital by an experienced team of pediatric gastroenterologists. Endoscopic findings in esophagus, stomach and duodenum were documented. Endoscopic features required for a diagnosis of EoE included esophageal mucosal furrowing, erythema, exudates, decreased vascular markings or circumferential rings. For the histopathological examination, four biopsies from the duodenum, two biopsies from the stomach, and three biopsies from both the proximal and distal esophagus were obtained with endoscopic biopsy forceps. The biopsy specimens were fixed immediately in formalin solutions for 4–6 h at room temperature and were routinely processed for conventional histological evaluation. All biopsies were reviewed with gastrointestinal pathologists with extensive experience with pediatric gastrointestinal pathology. Diagnosis of CD was based on the Modified Marsh classification of histologic findings. Marsh stage 1 biopsies are less specific and only considered to represent CD in the setting of positive (>10.0 U/mL) human recombinant tissue transglutaminase antibody IgA assay in symptomatic patients. EoE was diagnosed based on the presence of typical symptoms and an esophageal biopsy demonstrating greater than 15 eosinophils per HPF in the proximal and distal esophagus [2]. All patients with CD and EoE were referred to allergists for evaluation of food and environmental allergen sensitivities by skin prick test.

### Statistical analysis

All data is presented as mean ± standard deviation (SD). Student *t* test was used to compare the clinical characteristics in two groups of our study patients—those with CD and those with both CD and EoE. All statistical analyses were carried out using Statistical package for social science (SPSS) for Windows, version 18 (SPSS Inc., Chicago, IL, USA). Statistical significance was defined by *P* ≤ 0.05.

## Results

**Figure 1:**
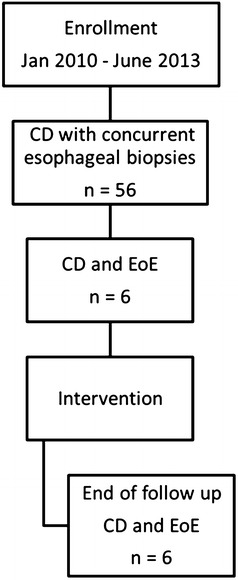
Distribution of patients. A total of 56 children were diagnosed with CD during the study period. Six patients were diagnosed with both CD and EoE. *CD* celiac disease, *EoE* eosinophilic esophagitis.

A total of 56 children were enrolled in the center during the study period, of whom fifty were diagnosed with CD. Six patients were diagnosed with both CD and EoE (Figure 1). The incidence of EoE in our study population was 10.7%. Table 1 outlines the characteristics and clinical presentation of the six patients identified with EoE and CD.

**Table 1:** The characteristics and clinical presentation of children with CD and EoE

Pt. no	Symptoms	Personal history	Family history	TTG IgA (U/mL)
1	Abdominal pain, diarrhea	Food allergies, type 1 diabetes mellitus	Type 1 diabetes mellitus	>300
2	Failure to thrive	Food allergies eczema, asthma	CD	112
3	Abdominal pain, diarrhea, increased flatulence	–	–	300
4	Abdominal pain, diarrhea, headache, mouth ulcers	–	CD	>300
5	Failure to thrive	–	–	>300
6	Abdominal pain, diarrhea, dysphagia	Food allergies	–	180

*CD* celiac disease, *EoE* eosinophilic esophagitis, *TTG IgA* anti-tissue transglutaminase antibody Ig.

The mean age of presentation in patients with both CD and EoE was 11.6 ± 1.6 years (mean ± SD). The mean duration of symptoms was 15.9 ± 2.2 months (mean ± SD). The majority of patients (5/6) were male. Most of our patients (4/6) presented with abdominal pain and diarrhea. Two patients reported failure to thrive alone, and dysphagia occurred in one patient. Food allergies were demonstrated in three patients based on skin prick testing. An abnormal celiac screening test (positive for human recombinant tissue transglutaminase antibody IgA) was seen in all six patients, and none was IgA deficient. All six patients were initially treated with empiric oral PPI (omeprazole) for GERD by primary care physicians at the time of study enrollment and biopsy. The median dose of omeprazole was 30 mg (range 20–40 mg) and median duration of use was 10 weeks (range 6–20 weeks).

The endoscopic and histopathological findings are described in Table 2. A variety of endoscopic findings were noted in all six patients. Endoscopic examination of the duodenum found mucosal changes that were suggestive of CD in four patients. Esophageal biopsies were taken from the proximal and distal esophagus in all six patients. None of the biopsy specimens from the stomach or duodenum showed significant eosinophilic infiltration. Esophageal fungal stain was negative, and there was no evidence of *Helicobacter pylori* histologically, or upon staining, in any patient. Most of the patients (4/6) with CD had a Marsh score of 3b, and two patients had a score of 2.

**Table 2:** Endoscopic, histopathological findings and treatment of children with CD and EoE

Pt. no	Endoscopy findings at diagnosis			Histopathological findings at diagnosis			Treatment	IEEo at followup	Total no of EGD performed
	Esophagus	Stomach	Duodenum	IEEo	Stomach	Duodenum (Marsh score)			
1	Linear furrowing and white exudates	Normal	Scalloping	24	Non specific gastritis	3b	Gluten-free diet, PPI, elimination diet	0	3
2	White exudates	Normal	Scalloping	30	Normal	2	Gluten-free diet, PPI, elimination diet	5	3
3	Linear furrowing	Normal	Normal	20	Normal	3b	Gluten-free diet, PPI	0	2
4	Linear furrowing and white exudates	Normal	Scalloping	>50	Normal	3b	Gluten-free diet, PPI	0	2
5	Linear furrowing and circumferential rings	Normal	Scalloping	>50	Non specific gastritis	3b	Gluten-free diet, PPI, swallowed fluticasone	2	4
6	White exudates	Normal	Normal	>50	Normal	2	Gluten-free diet, PPI, elimination diet, swallowed fluticasone	10	4

*CD* celiac disease, *EoE* eosinophilic esophagitis, *IEEo* intraepithelial esophageal count, *PPI* proton-pump inhibitor, *EGD* esophagogastroduodenoscopy.

Gluten-free diet was advised in all six patients, along with a PPI. PPI was continued after the diagnosis of EoE as a cotherapy to alleviate symptoms related to secondary GERD, which might be present with EoE [16]. Patients were evaluated again in the GI clinic within a 2–3-month period and a follow up endoscopy was performed in all subjects. We defined treatment response as resolution of endoscopic and histologic changes (<7 eosinophils per HPF with regression of basal layer hyperplasia and papillary lengthening) on follow-up endoscopy. Two patients (#3, #4) showed significant improvement in symptoms and resolution of endoscopic and histological changes with the gluten-free diet and a PPI. Three other patients (#1, #2, #6) were also treated with an elimination diet following confirmed food allergies. Two of these three patients (#1, #2) showed remarkable improvement in symptoms with resolution of eosinophilia at repeat esophageal biopsies on follow-up. Patient #6 didn’t improve and was managed with topical corticosteroid therapy [fluticasone dipropionate MDI (220 mcg), two puffs swallowed twice a day]. At the 6-month follow-up, the patient showed improvement in symptoms, and a repeat endoscopy revealed significant reduction in eosinophilic infiltration. One patient (#5) was also treated with topical corticosteroid therapy at the time of diagnosis. During follow-up, the patient showed resolution of eosinophilic infiltration at repeat esophageal biopsies, as well as clinical improvement.

We also compared the clinical characteristics in two groups of our study patients—those with CD and those with both CD and EoE. The results are given in Table 3. We noticed a significant difference in regards to gender and food allergies. Patients with CD and EoE were also noticed to have higher levels of TTG IgA in serum, although it didn’t reach statistical significance. Patients with CD and EoE were also noted to have higher Marsh scores and significant endoscopic changes in esophagus.

**Table 3:** Comparison of clinical characteristics between CD alone vs. with CD and EoE

Clinical characteristics	CD (n = 50) mean ± SD or n (%)	CD and EoE (n = 6) mean ± SD or n (%)	*P* value
Demographics			
Age at diagnosis (years)	10.5 ± 4.3	11.6 ± 1.6	0.536
Gender			
Male/Female	17 (34)/33 (66)	5 (83)/1 (17)	0.030
Race			
Caucasian/Other	48 (96)/2 (4)	5 (83)/1 (17)	0.293
Duration of symptoms (months)	13.8 ± 14.6	15.9 ± 12.2	0.738
History of allergies			
Asthma	2 (4)	1 (17)	0.293
Food allergy	8 (16)	4 (67)	0.016
Presenting symptoms			
Abdominal pain	27 (54)	4 (67)	0.682
Dysphagia	0 (0)	1 (17)	0.107
Failure to thrive	12 (24)	3 (50)	0.326
Diarrhea/Constipation	18 (36)	0 (0)	0.162
Endoscopy findings			
Normal esophagus	50 (100)	0 (0)	<0.0005
Ringed esophagus	0 (0)	1 (17)	0.035
Esophageal mucosal furrows	0 (0)	5 (85)	0.0005
White plaques in esophagus	0 (0)	4 (67)	0.001
Normal duodenum	24 (48)	4 (67)	0.669
Scalloping duodenum	25 (50)	2 (33)	0.671
Histopathological findings			
TTG IgA (U/mL)	164.4 ± 129.2	248.7 ± 82.4	0.058
Esophageal eosinophilia	1.1 ± 2.4	42 ± 20	0.0001
Marsh score			–
1	8 (16)	0 (0)	
2	8 (16)	2 (33)	
3a	16 (32)	0 (0)	
3b	18 (36)	4 (67)	

*CD* celiac disease, *EoE* eosinophilic esophagitis, *TTG IgA* anti-tissue transglutaminase antibody IgA, *SD* standard deviation.

## Discussion

Over the past few decades, both the incidence and prevalence of EoE in children increased significantly, which may be due to a genuine increase in incidence, greater recognition of EoE, or a higher use of diagnostic EGDs in children with the wide variety of symptoms suggestive of EoE [17–21]. Studies have shown that EoE can be associated with various other conditions including CD. The coexistence of EoE and CD in the same patient was first described in 2007 by Verzegnassi et al. [10], who observed that patients with EoE seemed more likely to develop CD than did the general population. Since then, a number of cases have been described in Italy by Quaglietta et al. [11], who found that in patients diagnosed with EoE, 35.2% had both diseases. This prevalence was lower (3.2%) in an Australian report of 221 children with CD, all of whom had undergone esophageal biopsies [12]. Leslie et al. [13] reported the prevalence of EoE to be 8.2% among 121 children with CD who had concurrent esophageal biopsies. A recent retrospective review also published increased incidence of EoE in both children and adults with CD compared to the general population [14]. The incidence of EoE in our cohort of children with CD was 10.7%.

The pathogenetic mechanism underlying the simultaneous presence of these conditions remains unknown. CD is an autoimmune enteropathy, thought to be mediated by Th1-immune response against ingested gluten-derived peptides in genetically susceptible individuals [22]. EoE, however, has been shown to be a Th2-mediated disease that is characterized by dense and isolated esophageal eosinophilia, which cannot be attributed to GERD or other causes. The underlying mechanisms of EoE remain unclear, but it is thought to be an immediate and delayed hypersensitivity disordered response to inhaled or ingested allergens. Interactions between genetic and environmental factors appear to be important. Familial patterns, usually male predominance (in the current study) and association with atopic conditions, have been described in previous studies [23].

It is unknown if gluten is the inciting antigen for esophageal eosinophilia in patients with CD. Studies have shown that a gluten-free diet alone failed to improve the manifestations of EoE in children with CD and EoE, suggesting involvement of antigens other than gluten. According to one theory [24], increased intestinal permeability secondary to CD may facilitate the exposure of the intestinal immune system to various antigens. Subsequent hypersensitivity reactions in genetically predisposed individuals at various body sites, including the esophagus, may lead to allergen sensitization, eosinophilic infiltration, and EoE. In our study, children with CD and EoE had higher serum levels of tissue transglutaminase IgA and moderate to severe villous atrophy in duodenal biopsies. Based on these findings, we can conclude that children with severe mucosal damage secondary to CD are at a higher risk of developing EoE, secondary to increased exposure to various antigens and allergens.

Immunologically, CD is a Th1-mediated disease, and EoE is a Th2-mediated immune response. The possibility of co-existing Th1 and Th2 diseases is still under debate. However, molecular studies have shown that autoimmune and atopic diseases share risk factors that increase the propensity of the immune system to generate both Th1 and Th2 mediated inappropriate responses to non-pathological antigens. These findings seem to suggest more than a casual association between CD and EoE and a more generalized defect of immune regulation [25–27].

Among adults with eosinophilic esophagitis, the predominant symptoms are dysphagia and food impaction. In a population-based epidemiologic study, these were also identified as the primary symptoms among older children and adolescents. Conversely, younger children and infants had different and vaguer symptoms, including vomiting and abdominal pain, and typically did not present with food impaction [28, 29]. Most of our patients did not present with symptoms suggestive of EoE; however, a significant proportion of patients were noticed to have food allergies. Regardless of their presenting symptoms, endoscopic changes were demonstrated in most of the patients, and microscopic changes were noticed in all of our patients.

It is unknown whether EoE in patients with CD responds to a gluten-free diet alone. Two patients in our study showed clinical improvement in symptoms with the gluten-free diet and a PPI, and this was confirmed by an upper endoscopy and histopathological examination. It is possible that these patients could have had PPI-REE; however, both patients received an adequate course of PPI at the time of endoscopy and biopsy. The lack of a clinicopathologic response to PPI treatment in these patients adherent to the treatment regimen with compatible symptoms of EoE and esophageal eosinophilia is consistent with the diagnosis of EoE. The rest of our patients required other forms of treatment including an elimination diet and oral corticosteroid treatment. Recent studies have shown that a gluten-free diet did not appear to induce remission of coexistent endoscopic and histopathological features of EoE in patients with CD. However, the sample size was smaller in this series, and these findings need to be confirmed with larger samples of patients [15].

We acknowledge several limitations of the present study. First, none of our patients had esophageal pH monitoring or impedance testing to rule out GERD as the cause of symptoms. Second, we studied comparatively few patients with CD and EoE, and the study may not adequately compare the clinical characteristics with the CD population of children. Third, there was no prolonged follow-up or outcome in our patients with CD and EoE, so it is unclear whether the various treatments used for EoE altered the natural history of this disease.

## Conclusion

Our study suggests that the incidence of EoE in our population of children with CD was 10.7%, which is higher than the previous reports. Our study confirms similar findings from various retrospective reviews—a higher than expected incidence of EoE compared with general population. We emphasize the importance of performing routine esophageal biopsies when investigating for CD, irrespective of the presenting symptoms and appearance of the esophageal mucosa at endoscopy. More prospective research is needed to determine the effect of various treatment modalities on the outcome of EoE.

## Abbreviations

CD: celiac disease
EoE: eosinophilic esophagitis
HPF: high power field
GERD: gastroesophageal reflux disease
PPI: proton-pump inhibitor
PPI-REE: proton-pump inhibitor-responsive esophageal eosinophilia
SD: standard deviation
SPSS: statistical package for social science
IEEo: intraepithelial esophageal count
TTG IgA: anti-tissue transglutaminase antibody IgA
EGD: esophagogastroduodenoscopy
